# Clinical phenotype of the 16p.13.11 microdeletion: a case report with a mini review of the literature

**DOI:** 10.3389/fgene.2024.1429185

**Published:** 2024-08-16

**Authors:** Roberto Palumbi, Emanuela Ponzi, Stefania Micella, Mara Pascali, Roberta Bucci, Mattia Gentile, Lucia Margari, Marta Simone

**Affiliations:** ^1^ Child Neuropsychiatry Unit, Department of Translational Biomedicine and Neurosciences–DiBraiN, University of Bari Aldo Moro, Bari, Italy; ^2^ Medical Genetics Unit, Bari, Italy; ^3^ Child Neuropsychiatry Unit, Department of Precision and Regenerative Medicine and Jonic Area, University of Bari Aldo Moro, Bari, Italy

**Keywords:** case report, chromosome 16p13.11 microdeletion, copy number variations, neurodevelopment, autism spectrum disorder

## Abstract

**Background:**

Chromosome 16p13.11 microdeletion is a very rare copy number variant (CNV), associated with a clinical syndrome characterized by global development delay, neuropsychiatric conditions, facial dysmorphisms, microcephaly, gastroesophageal reflux disease, and congenital heart defects. The 16p13.11 locus is a very unstable genomic region, rich in low-copy number repeats, characterized by many homologous DNA sequences. Usually, the most common CNV of this region include microduplications/duplications, while the microdeletions are rare, and their clinical features are heterogeneous and poorly described so far.

**Case report:**

In this paper, we report the genetic and the clinical features of a patient diagnosed with chromosome 16p13.11 microdeletion, and a short review of the literature on this topic. Our patient was characterized by several facial dysmorphic features, autistic symptoms and language development delay. The genetic evaluation revealed and interstitial deletion of the long arm of the chromosome 16, approximately of 1.5 Mb.

**Conclusion:**

Interestingly, compared to previous cases, this patient was characterized by autistic symptoms, severe language and motor coordination disorder, without cognitive and cerebral malformations, frequently associated with this microdeletion syndrome.

## 1 Introduction

Chromosome 16p13.11 microdeletion is a rare copy number variant (CNV), associated with a clinical syndrome characterized by motor delay, facial dysmorphisms, microcephaly, gastroesophageal reflux disease (GERD), and congenital heart defects (Granata et al., 2022).

Structural variation of the human genome, including deletions, duplications and insertions result in CNV. The clinical impact of CNVs is often complicated by marked clinical heterogeneity and incomplete penetrance; genotype-phenotype correlation studies are needed to better understand the clinical significance of CNVs, like the rearrangement in 16p13.11 chromosomal region (Atli et al., 2022; Granata et al., 2022).

The most common 16p13.11 microdeletions identified so far usuallyrange from 0.8 to 3.3 Mb (Martin et al., 2004; Ullmann et al., 2007; Hannes et al., 2009; Nagamani et al., 2011; Paciorkowski et al., 2013; Miteff et al., 2015; Tan et al., 2017; Atli et al., 2022). Among other genes, this region includes a core set of eight protein-coding genes, as the one coding for the Nude Neurodevelopment protein 1 (*NDE1*), which is the strongest candidate gene for the neurodevelopmental phenotypes associated with the 16p13.11 microdeletions (Tan et al., 2017; Granata et al., 2022). *NDE1* gene encodes for the “nuclear distribution protein nudE homolog 1”, a centrosomal protein that has a crucial role in the process of mammalian brain development, specifically in the human cerebral cortex growth. The N-terminal Asparagine Amidase 1 (*NTAN1*) is another important candidate gene located in the 16p13.11 region. This gene has a sequence highly conserved throughout the species evolution (Tan et al., 2017; Granata et al., 2022). For example, in *Drosophila*, *NTAN1* protein isoforms share a large part of the sequences when compared with the human versions. In addition, it is expressed in several organs, including the brain. This protein has been associated with social/behaviour alterations in mice models and with cancer and schizophrenia in humans (Castañeda-Sampedro et al., 2022). However, its role in the human brain development is unknown.

About the 16p13.11 microdeletion syndrome, the management of the patients during the neonatal period includes not only psychomotor development alterations, but also the associated comorbidities, including feeding problems and the gastroesophageal reflux disease. Life span implications of chromosome 16p13.11 microdeletion syndrome includes several neuropsychiatric conditions, as global developmental delay, psychiatric and behavioral problems, seizures and, less commonly, obesity. In addition, nursing assessment is globally crucial for the early identification of non-specific abnormalities associated with *de novo* syndrome (Nagamani et al., 2011; Atli et al., 2022).

In this paper, we present a case report of an infant diagnosed with chromosome 16p13.11 microdeletion and a short review of the previous reported cases.

A discussion of genetic influences associated clinical manifestations, diagnostics, management, and health promotion strategies are presented to establish core knowledge of chromosome 16p13.11 microdeletion.

## 2 Case description

### 2.1 Clinical features

The informed consent for this publication was collected by the child’s parents. Our patient is a 4-year-old boy born with natural full-term birth by non-consanguineous Italian father and Romanian mother. He is a single-born and the pregnancy was undisturbed. At birth, his weight was 3.420 kg. No respiratory distress signs were referred. The motor development was normal; he walked autonomously by 18 months. The language development was very compromised and slow; at 3 years old, the boy spoke using holophrases and he knew only few words with poor subsequent improvements. The patient was bottle-fed and weaning progressed without difficulty in adapting to the various consistencies. However, at the age of 3, nutrition becomes more selective (he preferred exclusively yogurt, milk and rarely solid food). The language difficulties and the atypical behaviour were the reasons for a neuropsychiatric counselling. The patient was admitted to our Child Neuropsychiatry Division of the University Hospital of Bari, Italy. During the hospitalization, he underwent a complete neuropsychiatric assessment. The Leiter Test (Lewis et al., 2013) was used to assess non-verbal intelligence, showing an Intelligence Quotient of 111. The mother reported the presence of restricted and repetitive patterns of behaviour. The Autism Diagnostic Observation Schedule–2– Module (Lord et al., 2012) was performed; his total score was 4, assessing a low-grade severity of autistic symptoms. The behavioural observation revealed motor stereotypies (hand flapping), echopraxia and echolalia.

The general examination revealed the presence of the following dysmorphic features: hypertelorism, bilateral mild epicanthus, nose root hypoplasia, ligamentous hyperlaxity, II-III toes syndactyly bilateral. Furthermore, he held his mouth mostly open with a cupid bowed upper lip and he showed a full lower lip with a slightly protruding tongue.

To better investigate the patient’s condition, a genetic counselling was performed in collaboration with the Genetic Unit of Di Venere Hospital of Bari. The karyotype performed was normal. The Array-CGH genetic test revealed the presence of 16p13.11 microdeletion, extended for 1.5 Mb and involving 12 genes; the genetic evaluation of the parents revealed that this microdeletion was inherited by his father.

Moreover, the following investigations were performed: 1) a brain magnetic resonance Imaging (MRI) reported these results: ventricular system on axis, of regular size. Multiple minute areas of hypersignal in the T2-weighted sequences are observed scattered in the subcortical white matter of both cerebral hemispheres, of non-specific significance. The cortical tape appears to be regular; 2) cardiologic counselling and a colour doppler echocardiography reported no clinical and instrumental signs of heart disease; 3) a complete abdomen ultrasound resulted normal; 4) the electroencephalogram showed a normal cortical electric activity.

### 2.2 Genetic evaluation

Cytogenetic analysis was performed on QFQ-banded metaphases (550-band level): in all the examined metaphases, a normal male karyotype (46,XX) was observed. Genomic DNA was extracted from peripheral blood samples. DNA concentration was measured by fluorimeter (Amersham, Piscataway, NJ) using the Hoechst reagent and adjusted to 400 ng/mL. Array-CGH analysis was performed using the Cytochip oligo ISCA 4 × 180K (TechnoGenetics Srl). The analysis revealed an interstitial deletion involving the long arm of chromosome 16: arr [GRCh37] 16p13.11 (14868192_16400833)x1, size 1.5 Mb. Segregation analysis showed paternal origin of microdeletion. The 1.5 Mb deleted region contains 12 OMIM genes: *NOMO1*(*609157), *NPIPA1*(*606406), *PDXDC1* (*614244), *NTAN1* (*615367), *RRN3* (*605121), *MPV17*(*618100), *NDE1* (*609449), *MYH11*(*160745), *FOPNL* (*617149), *ABCC1*(*158343), *ABCC6*(*603234), *NOMO3*(*609159). Therefore, the final karyotype of the patient was designed as (ISCN 2016): 46,XX.arr [GRCh37] 16p13.11 (14868192_16400833)x1 pat.

## 3 Discussion

In this case report, we describe the clinical features of a 4-years old boy carrying a 1.5 Mb microdeletion in the 16p13.11 chromosomal region, a rare genetic CNV, characterized by incomplete penetrance and highly variable phenotype expression. Few cases are reported in the literature with this genetic alteration associated with multiple body organs malformations (e.g., congenital heart defects, gastrointestinal malformations), but also with neurodevelopmental and/or neurological conditions, as global development delay, epilepsy, autism and other behavioral problems (Ullmann et al., 2007; de Kovel et al., 2010; Heinzen et al., 2010; Mefford et al., 2010; Nagamani et al., 2011; Kloth et al., 2019; Atli et al., 2022; Tropeano et al., 2013).

As far as we can learn, the clinical outcomes of patients affected by 16p13.11 microdeletion are quite variable and unpredictable. The diagnosis of this rare condition might be challenging even from the neonatal period (Mefford et al., 2010). Its early detection is crucial for the management of some complications, including congenital heart defects and/or feeding difficulties, alongside with the neurological and neurodevelopment implications (Mefford et al., 2010; Smith et al., 2018). Moreover, the prenatal diagnosis might be difficult as well, considering that specific features related to this microdeletion are rarely described (Law et al., 2009; Cai et al., 2022; Kang et al., 2023). Cai et al. retrospectively explored the potential association between prenatal ultrasound characteristics of fetuses affected by 16p13.11 microdeletion/microduplication, showing no significant ultrasound intrauterine features of these fetuses (Cai et al., 2022).

The patient features compared with other patients previously described are reported in Table 1.

**TABLE 1:** Comparison between our patient and previously described cases presenting mostly neurological and/or neurodevelopment implications.

Case	Gender	Genetic features	Clinical phenotype	Brain MRI/TC
Our patient	Male	1.5 Mb microdeletion of 16p13.11 involving 12 genes	Dysmorphic features, autistic symptoms, language development impairment	Normal
Case 1 Balasubramanian et al. (2011)	Male	0.16–2.41 Mb deletion in the 16p13.11 region, including 12 genes	Microcephaly, severe global develooment delay, West Syndrome, facial dysmorphic features	Global hypomyelination
Case 2 Paciorkowski et al. (2013)	Male	0.83 Mb deletion of 185 probes on 16p13.11	Dysmorphic features, severe developmental delay, epilepsy, body temperature instability	Agenesis of the corpus callosum, increased extra-axial space, a cortical dysplasia polymicrogyria-like, and a disproportionately small brainstem and cerebellum
Case 3 Paciorkowski et al. (2013)	Male	Deletion of five markers flanking NDE1 on 16p13.11	Dysmorphic features, language development delay, one episode of seizures, body temperature instability	Agenesis of the corpus callosum, a disproportionately small brainstem, and a large interhemispheric fluid space adjacent to the right lateral ventricle
Case 4 Miteff et al. (2015)	Female	1.34 Mb microdeletion of 16p13.11 involving 13 genes	Hemiconvulsion-hemiplegia-epilepsy syndrome	Right emisphere atrophy
Case 5 Tan et al. (2017)	Male	16p13.11 deletion containing NDE1 gene, and a novel NDE1 mutation c.555_ 556 GC > CT	Severe microcephaly, global developmental delay	Agenesis of the corpus callosum, enlargement of the posterior lateral ventricles, and a simplified gyral pattern of the cerebral cortex with scarce and wide gyri in parietal and occipital lobes
Case 6 Maldžienė Ž. et al. (2019)	Female	2.5 Mb deletion in the 16p3.11-p12.3 genomic region, involving 21 genes	Developmental delay, sagittal craniosynostosis, minor facial anomalies	Brain TC (at the age of 3 months): Dolichocephaly, disproportionately prologed, parietal bones, sagittal craniosynostosis, mild asymmetry of the lateral ventricles, and hypoplasia of the cerebellar vermis. Brain MRI (at the age of 3 years): enlarged cisterna magna and subcortical nonspecific small gliotic focus in the left frontal lobe

In 2011, Balasubramanian et al. (2011) described a patient characterized by severe global development delay, microcephaly, infantile spasms (later associated with a West Syndrome), feeding problems from his birth, and some body and facial dysmorphism. Array-CGH found an interstitial deletion in 16p13.11 region, between 0.16 and 2.41 Mb in size, including 12 genes. In 2013, Paciorkowski et al. (2013) reported two cases of not-related patients affected by brain malformations, such as severe microcephaly, agenesis of the corpus callosum, scalp rugae, and a fetal brain disruption (FBD)-like phenotype with inherited deletions of 16p13.11. In 2015, Miteff et al. (2015) described for the first time a clinical case of a patient affected by a 16p13.11 microdeletion associated with an hemiconvulsion-hemiplegia-epilepsy syndrome, suggesting the hypothesis that this genetic alteration might affect the brain development in association with a susceptibility to an epileptic syndrome. Later, other authors described a case of an 8-month patient carrying the same genetic alteration, involving the NDE1 gene. He was characterized by a severe congenital microcephaly and the neuroimaging procedures showed an agenesis of the corpus callosum, enlargement of the posterior lateral ventricles, and a simplified gyral pattern of the cerebral cortex (Tan et al., 2017). In 2019, Maldžienė et al. (2019) reported a case of a 4 years and 7 months girl affected by minor facial anomalies, mild neurodevelopmental delay and a sagittal craniosynostosis, and carrying a 2.6-Mb deletion in the 16p13.11-p12.3. In a recent study, the authors described the clinical phenotype associated with CNV of chromosome 16 in a cohort of 18 patients, reporting in three patients only duplications of the 16p13.11 region with phenotypes ranging from ASD to craniosynostosis (Atli et al., 2022).

Interestingly, compared with these previous cases, our patient was characterized by neurodevelopment and behavioural disorders rather than neurologic conditions, without brain malformations, already described in previous cases.

Chromosome 16p13.11 locus is rich of segmental copies of genes and this feature makes this chromosome at high risk for genomic instability and very vulnerable for non-allelic rearrangement leading to variable types of CNV (Martin et al., 2004). Deletions of another chromosome 16 region, the 16p11.2 locus, are frequently reported in association with ASD and other neurodevelopment conditions, seizures and facial malformations (Atli et al., 2022). Using Whole Genome Sequencing (WES), a recent original research studied several variants of genes involved in neurodevelopment from SFARI database, and genes mapped in the region 16p13.11 in four patients carrying a 16p13.11 microdeletion (Granata et al., 2022). As a whole, variants in candidate genes of interest were identified in this region, including *NDE1* and *NOMO1* genes, all potentially involved in the clinical phenotype of the patients investigated (Alkuraya et al., 2011; Paciorkowski et al., 2013; Granata et al., 2022; Zhang et al., 2024). As previously described, *NDE1* gene encodes for the Nuclear Distribution Factor E-homolog 1 (nudE), a protein involved in several neuronal development processes, as neuronal mitosis and migration, cortex growth and organization (Pawlisz et al., 2008; Tan et al., 2017). Moreover, *NDE1* has been postulated to be responsible for microcephaly that is often seen in those with a 16p13.11 microdeletion (Alkuraya et al., 2011).

*NOMO1* is a negative regulator of the Nodal signaling pathway, involved in the formation of the neuroectoderm and mesoderm during the embryogenesis. A recent study showed that adult Nomo1-deficient zebrafish was characterized by autistic traits as social deficits, repetitive stereotypic behaviors and hyperactivity.

On the whole, all the data included in this case report, highlight not only the importance of this genomic region and its CNVs in the brain development, but also the high phenotypic variability associated with the microdeletions of this genomic region. Therefore, a careful genetic counseling targeting chromosome 16 CNV should be considered in cases of neurodevelopment disorders in patients with or without dysmorphic conditions, or other multiorgan abnormalities.

## Data Availability

The datasets presented in this article are not readily available because it is a single case description. Requests to access the datasets should be directed to MS.
